# Clinical case of lung spindle cell carcinoma markedly responsive to pembrolizumab

**DOI:** 10.1111/1759-7714.14068

**Published:** 2021-07-05

**Authors:** Yoshiko Mizushina, Fumiyoshi Ohyanagi, Jun Shiihara, Motoko Nomura, Hiromitsu Ohta, Hisashi Oshiro, Hiroyoshi Tsubochi, Gen Kusaka, Yasuhiro Yamaguchi

**Affiliations:** ^1^ Division of Pulmonary Medicine, Saitama Medical Center Jichi Medical University Saitama Japan; ^2^ Division of Pulmonary Medicine, Department of Medicine Jichi Medical University Tochigi Japan; ^3^ Thoracic Oncology, Saitama Cancer Center Saitama Japan; ^4^ Department of Pathology, Saitama Medical Center Jichi Medical University Saitama Japan; ^5^ Division of Thoracic Surgery, Saitama Medical Center Jichi Medical University Saitama Japan; ^6^ Department of Thoracic Surgery Jichi Medical University Tochigi Japan; ^7^ Department of Neurosurgery, Saitama Medical Center Jichi Medical University Saitama Japan

**Keywords:** immune checkpoint inhibitor, lung cancer, pembrolizumab, pseudoprogression, spindle cell carcinoma

## Abstract

A 52‐year‐old man underwent pneumonectomy of the left lung for previously diagnosed primary spindle cell carcinoma (pT4aN1M0, stage III B) with programmed death‐ligand 1 expression (tumor proportion score ≥95%) and without epidermal growth factor receptor gene mutation and anaplastic lymphoma kinase fusion gene. However, brain metastasis and chest wall tumor relapse occurred. Considering insufficient improvement with gamma knife treatment for brain metastasis and combination chemotherapy (paclitaxel, carboplatin, and bevacizumab), pembrolizumab monotherapy and palliative irradiation therapy for chest metastases were started after brain tumor volume reduction using craniotomy. Brain edema and chest wall metastases markedly improved following a pseudoprogression of the brain edema accompanied by a performance status decline; this effect continued until 11 cycles of pembrolizumab administration.

## Graphical abstract

Lung spindle cell carcinoma has a poor prognosis and is usually resistant to cytotoxic chemotherapy. Our findings suggest that immune checkpoint inhibitors (ICIs) are a novel therapeutic strategy for lung spindle cell carcinoma.

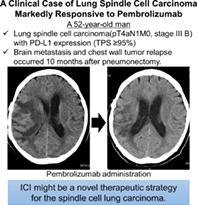

## CASE REPORT

**FIGURE 1:**
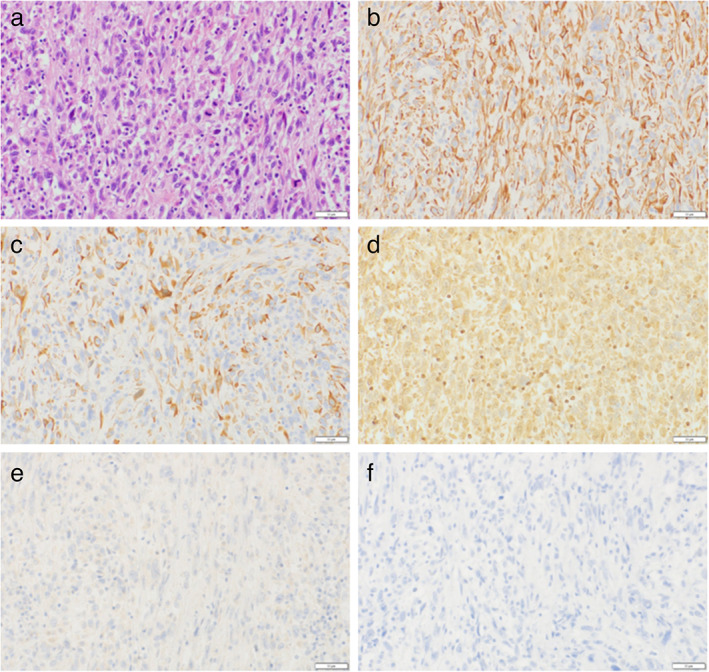
Histopathological findings of the pulmonary spindle cell carcinoma. (a) The neoplastic cells consist of an almost pure population of atypical spindle cells without differentiation (hematoxylin and eosin stain, the bar = 50 μm). (b) The neoplastic cells are positive for pan‐cytokeratin (AE1/AE3) (immunohistochemistry, the bar = 50 μm). (c) The neoplastic cells are positive for cytokeratin 7 (immunohistochemistry, the bar = 50 μm). (d) The neoplastic cells are weakly positive for vimentin (immunohistochemistry, the bar = 50 μm). (e) The neoplastic cells are negative for p40 (immunohistochemistry, the bar = 50 μm). (f) The neoplastic cells are negative for thyroid transcription factor‐1 (immunohistochemistry, the bar = 50 μm)

**FIGURE 2:**
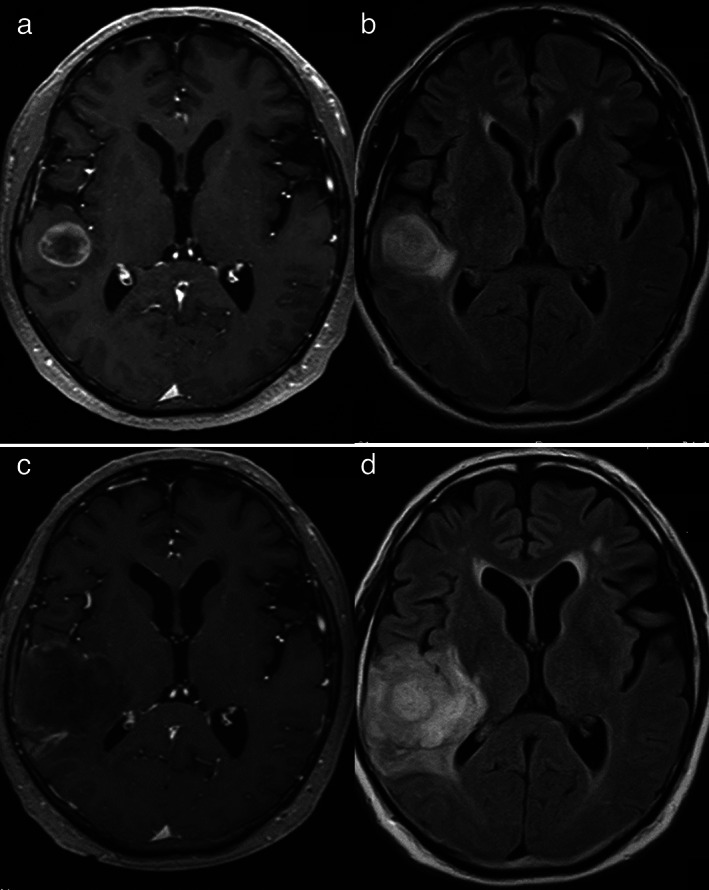
Brain magnetic resonance imaging nine and 14 months after pneumonectomy. (a) A brain metastasis in the right temporal lobe nine months after pneumonectomy (T1‐weighted image (WI) with gadolinium (Gd) enhancement). (b) Brain edema around the tumor nine months after pneumonectomy (fluid‐attenuated inversion recovery [FLAIR]). (c) Increase in brain tumor size 14 months after pneumonectomy (T1‐WI with Gd enhancement). (d) Worsening perifocal edema 14 months after pneumonectomy (FLAIR)

A 52‐year‐old man, who smoked 34 packs/year and was an ex‐smoker, was diagnosed with a left lower lobe lung tumor and subsequently underwent pneumonectomy of the left lung. He was diagnosed with primary lung spindle cell carcinoma (SpCC; pT4aN1M0, stage III B), with programmed death‐ligand 1 (PD‐L1) expression (tumor proportion score [TPS] ≥95%) without epidermal growth factor receptor gene mutation and anaplastic lymphoma kinase fusion gene (Figure 1). Brain metastasis in the right temporal lobe was observed nine months after pneumonectomy despite the patient receiving four cycles of cisplatin and vinorelbine as postoperative adjuvant chemotherapy (Figure 2(a),(b)). Brain metastasis was treated with gamma knife surgery. However, radiation necrosis was suspected because no reduction was observed in the tumor size and perifocal edema. Moreover, progressively increasing chest wall metastases appeared 10 months after pneumonectomy. Despite combination chemotherapy (paclitaxel, carboplatin, and bevacizumab) given as first line chemotherapy 14 months after pneumonectomy, his brain edema progressively worsened (Figure 2(c),(d)). Thus, craniotomy and brain tumor volume reduction were performed, with pathological examination revealing metastatic lung carcinoma with necrosis.

**FIGURE 3:**
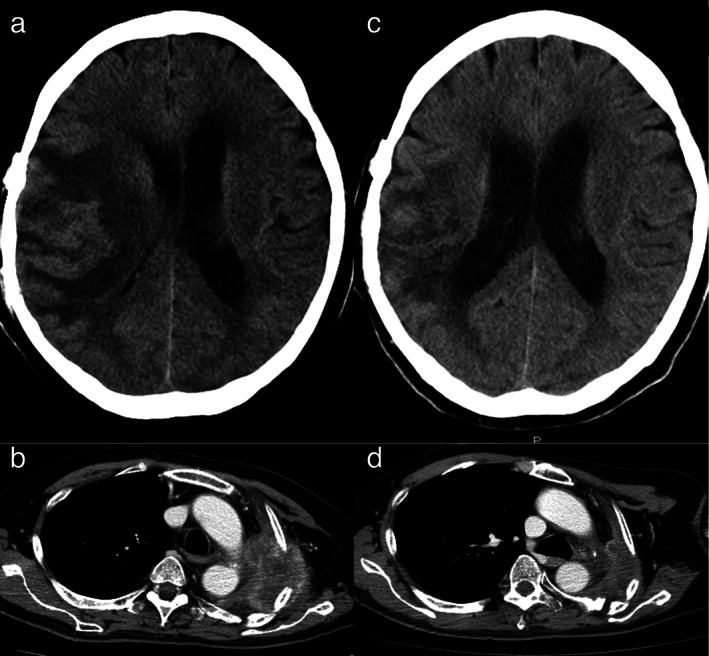
Brain and chest computed tomography (CT) before and after pembrolizumab administration. (a) Brain CT on Day 8 of the first pembrolizumab monotherapy cycle. Brain edema is markedly exacerbated in the right temporal lobe with midline shift. (b) Chest CT after two cycles of pembrolizumab. Chest wall tumors increased in size before initial pembrolizumab administration. (c) Brain CT after nine cycles of pembrolizumab. Brain edema has markedly decreased. (d) Chest CT after nine cycles of pembrolizumab. The chest wall tumor has markedly decreased in size

Throughout that period, chest wall metastases progressively increased in size; the patient had a performance status (PS) score of 2. Pembrolizumab monotherapy (200 mg/bodyweight, every three weeks) was started as second‐line chemotherapy one month after craniotomy. Palliative irradiation therapy (30 Gy/10 fr) to the chest wall was also performed from Day 3 after pembrolizumab monotherapy initiation. From Day 1, he developed a severe fever and exhibited appetite loss, general fatigue, and consciousness impairment (drowsy state) (PS score increased to 4). On Day 8, brain computed tomography (CT) showed an exacerbation of the brain edema (Figure 3(a)). Given the lack of improvement in fever despite antibiotic treatment, the fever was suspected to be associated with the tumor or reactive against pembrolizumab. To control the fever, naproxen was administered. The fever and consciousness level gradually improved, and he was discharged on Day 30.

After two cycles of pembrolizumab monotherapy, the patient's appetite and PS began to improve. Chest CT showed larger chest wall tumors than those after initial pembrolizumab administration (Figure 3(b)). His PS score improved to two after five cycles, and chest and brain CT showed reduction in the chest wall tumor size and brain edema (Figure 3(c),(d)).

After 12 and 15 cycles of pembrolizumab, brain magnetic resonance imaging showed new brain metastasis and chest wall tumor regrowth. Therefore, pembrolizumab monotherapy was stopped, and another round of gamma knife treatment for the brain metastases was performed. Despite recommending cytotoxic chemotherapies, the patient refused and died four months after pembrolizumab monotherapy.

## DISCUSSION

This patient was diagnosed with lung SpCC via surgical tumor resection but developed recurrence nine months after pneumonectomy. The recurrent lesions were refractory to irradiation and chemotherapy (cytotoxic chemotherapy and antiangiogenic treatment). In 2015, the World Health Organization classified pulmonary SpCC as a sarcomatoid carcinoma, comprising an almost pure population of epithelial spindle cells, without differentiated carcinomatous elements. Sarcomatoid carcinomas are rare (<1% of all lung cancers), have a poor prognosis, and are resistant to cytotoxic chemotherapy.^1^, ^2^

In the current case, the resected tumor showed high PD‐L1 expression and a significant therapeutic response to pembrolizumab. Sarcomatoid carcinomas, including the sarcomatous area of pleomorphic carcinomas, show high PD‐L1 expression and have poor prognosis.^3^, ^4^ Pembrolizumab, an immune checkpoint inhibitor (ICI), is a highly selective, humanized, immunoglobulin G4 monoclonal antibody against programmed cell death‐1.^5^ Despite the proven efficacy of pembrolizumab monotherapy against PD‐L1‐expressing non–small cell lung cancer (NSCLC) with a TPS of ≥1%, information regarding its effects against lung SpCC is scarce.^5^ Meanwhile, pembrolizumab monotherapy effectively decreased lung and metastatic tumors in a patient with SpCC.^6^ Furthermore, ICIs showed effects against sarcomatoid lung carcinoma and mesothelioma.^7^, ^8^

The current case developed brain metastases, resistant to irradiation and exhibited radiation necrosis. Patients with NSCLC who develop brain metastases have a poor prognosis and survival.^9^, ^10^ As metastatic brain tumors are protected by the blood–brain barrier (BBB), ICI efficacy remains unclear. BBB is often compromised in patients with brain metastases and is subsequently remodeled into a blood–tumor barrier, altering the immune cell population around brain metastases.^9^, ^11^, ^12^ Moreover, ICIs possibly exert a suppressive effect on the progression of central nervous system tumors despite no established evidence that demonstrate the efficacy of ICI against brain metastases.^9^, ^10^, ^11^, ^12^, ^13^, ^14^, ^15^ Therefore, pembrolizumab penetration into the brain tumor was possible in this case because BBB was disrupted by the preceding brain tumor resection.

In the current case, brain edema exacerbated with PS decline, following initial pembrolizumab administration. Initial ICI treatment for brain metastases often shows a pseudoprogression within the first three months.^9^ Pseudoprogression of brain metastases has been explained histologically by inflammatory cell infiltration, edema, and necrosis, and radiologically by frequent exhibition of perilesional brain edema.^16^, ^17^, ^18^ A careful therapeutic response evaluation is thus recommended to avoid progressive disease six months or less after ICI administration initiation.^9^, ^19^ In retrospect, brain edema and chest wall tumor exacerbation after initial pembrolizumab administration may suggest a pseudoprogression given the remarkable therapeutic effect observed thereafter. Although it is difficult to distinguish the contribution of abscopal effect by palliative irradiation therapy to chest wall, exacerbation of brain edema could be explained adequately by pseudoprogression of ICI.^16^, ^17^, ^20^ Although brain edema exacerbation frequently induces PS decline, a pseudoprogression would usually exhibit a subsequent improvement. Therefore, a pseudoprogression should be considered to make appropriate decisions regarding ICI administration continuation.

Lung SpCC has a poor prognosis and is usually resistant to cytotoxic chemotherapy. Our findings suggest ICI therapy as a novel therapeutic strategy for lung SpCC. Furthermore, given that pseudoprogression with PS decline is commonly observed in brain metastases treated with ICIs, the therapeutic response should be carefully evaluated to determine whether ICI can be continued.

## CONFLICT OF INTEREST

No authors report any conflict of interest.
